# Effectiveness of Vitamin D on Neurological and Mental Disorders

**DOI:** 10.3390/diseases12060131

**Published:** 2024-06-20

**Authors:** Shareefa Abdullah AlGhamdi

**Affiliations:** 1 Department of Biochemistry, Faculty of Sciences, King Abdulaziz University, Jeddah 21589, Saudi Arabia; saaalghamdi1@kau.edu.sa; Tel.: +966-506-352-828; 2 Vitamin D Pharmacogenomics Research Group, King Abdulaziz University, Jeddah 21589, Saudi Arabia; 3 King Fahd Medical Research Center, King Abdulaziz University, Jeddah 21589, Saudi Arabia

**Keywords:** vitamin D, mental disorders, depression, epilepsy, bipolar, schizophrenia

## Abstract

(1) Background: Mental disorders are conditions that affect a person’s cognition, mood, and behaviour, such as depression, anxiety, bipolar disorder, and schizophrenia. In contrast, neurological disorders are diseases of the brain, spinal cord, and nerves. Such disorders include strokes, epilepsy, Alzheimer’s, and Parkinson’s. Both mental and neurological disorders pose significant global health challenges, impacting hundreds of millions worldwide. Research suggests that certain vitamins, including vitamin D, may influence the incidence and severity of these disorders; (2) Methods: This systematic review examined the potential effects of vitamin D supplementation on various mental and neurological disorders. Evidence was gathered from databases like PubMed, Cochrane, and Google Scholar, including multiple randomized controlled trials comparing vitamin D supplementation to placebo or no treatment for conditions like depression, bipolar disorder, epilepsy, schizophrenia, and neuroinflammation; (3) Results: The findings strongly indicate that vitamin D supplementation may benefit a range of mental health and neurological disorders. The magnitude of the beneficial impact varied by specific disorder, but the overall pattern strongly supports the therapeutic potential of vitamin D on these disorders; (4) Conclusions: This review provides valuable insight into the role vitamin D may play in the management of critical brain-related health issues.

## 1. Introduction

Mental health disorders are among the issues affecting the global population [1]. According to the recent report published in 2023 by the World Health Organization (WHO), it is estimated that approximately 3.8% of the global population experience depression. This includes 5% of adults, with a higher rate among women (6%) compared to men (4%). The prevalence is even higher among older adults, with 5.7% of those over 60 years old affected by depression. In total, it is estimated that around 280 million people worldwide have depression. The condition is about 50% more common in women than in men. The impact of depression is particularly acute during major life events. Over 10% of pregnant women and new mothers experience depression. Depression also has tragic consequences as it is a leading contributor to suicide. More than 700,000 people die by suicide globally each year, making it the fourth leading cause of death among 15–29-year-olds. These statistics underscore the substantial global burden of depression, which affects hundreds of millions of people across all ages and demographics. Addressing this major public health issue remains a critical priority worldwide [2]. Different researchers have attempted, through various strategies, both medical and non-medical, to enable the prevention and treatment of these disorders [3,4,5]. According to WHO, neurological disorders have a significant global impact, affecting hundreds of millions of people worldwide. Each year, stroke alone claims the lives of over six million people, with more than 80% of these fatalities occurring in low- and middle-income countries. Epilepsy affects more than 50 million people worldwide, while the estimated global count of individuals with dementia is around 47.5 million, with an additional 7.7 million new cases reported annually. Alzheimer’s disease, the leading cause of dementia, is responsible for approximately 60–70% of cases. The occurrence of migraines, with a prevalence exceeding 10% worldwide, is also a widespread neurological condition [6]. Vitamin supplementation has gained more scientific attention based on its implications and effects on different mental health and neurological disorders [7,8]. Numerous researchers have noted the effects of the vitamin B complex in boosting mental health, while others have featured the relevance of vitamin D, Omega-3, and amino acids [9,10]. Regardless, efficacy is majorly based on a generalised aspect. Specific vitamin supplements have shown more effectiveness than other supplements, which are also based on a particular mental health disorder. There is considerable concern regarding the potential impact of insufficient levels of vitamin D on the progression of diseases. There is a growing body of research suggesting a link between vitamin D and depression, as well as other psychological disorders. Thus, abundant research studies have investigated the effectiveness of vitamin D supplements in the treatment of mental and neurological disorders [11,12,13,14]. From this viewpoint, vitamin D receptors are present in various crucial brain regions [15]. Within these areas, vitamin D appears to play a role in the development and maturation of neurons, the regulation of growth factor production (including neural growth factor and glial cell line-derived growth factor), and the synthesis of various neurotransmitters, including acetylcholine, dopamine, and gamma-aminobutyric acid [15]. Vitamin D is categorized as being part of the neuroactive steroid group of compounds due to the observed associations between vitamin D deficiency and several neuropsychiatric disorders [16]. These neuropsychiatric conditions include Alzheimer’s disease, autism, depression, and schizophrenia. Interestingly, in a recent study, it was found that a post-COVID cohort presenting long-term neurocognitive impairment had a lower baseline and follow-up 25-hydroxyvitamin D levels compared to those without such an impairment, possibly further confirming the well-known influence of vitamin D in this specific health domain [17]. Hence, vitamin D is among the most crucial supplements that have been suggested to be an effective alternative option in preventing and treating mental health disorders [8,18]. However, these studies have generated conflicting results, with some suggesting that vitamin D supplementation may be beneficial for improving symptoms of depression, while others have found no significant effect. Similarly, studies investigating the effects of vitamin D supplementation on neuroinflammation, schizophrenia, and epilepsy have also reported inconsistent results. The primary biomarker used to assess vitamin D status is the serum concentration of 25-hydroxyvitamin D [25(OH)D], which reflects both dietary intake and the cutaneous synthesis of the vitamin. An adequate 25(OH)D level is generally considered necessary to support diverse physiological processes and minimize the risk of deficiency-related disorders [19]. The primary objective of this systematic review was to comprehensively evaluate the existing research on the effect of vitamin D supplementation on various mental disorders, including depression and bipolar, as well as neurological disorders such as epilepsy, schizophrenia, and neuroinflammation. The selection of specific disorders was based on several factors. Firstly, these disorders were chosen because they have been extensively mentioned in local studies and are commonly discussed within the Middle Eastern community [20,21]. This ensures that the research aligns with the prevailing concerns and issues faced by individuals in the Middle Eastern cultural context [22]. Moreover, these disorders were selected because they tend to be underdiagnosed due to the social stigma surrounding open discussions about mental and neurological health [23]. By focusing on these disorders, the study aims to shed light on their prevalent impact and potential treatment options, thereby contributing to breaking down the barriers and pushing people to take care of their health overall. Additionally, the inclusion of neurotypical disorders such as neurological inflammation and epilepsy was justified by their higher occurrence within the Middle Eastern culture [24,25]. In Saudi Arabia, the prevalence of mental and neurological disorders mirrors global trends [26]. Hence, by conducting studies on these specific disorders, it is possible to generate valuable insights that can encourage people to prioritize their health, including the adoption of vitamin D supplementation. This would help clarify the therapeutic potential of vitamin D for these widespread and often debilitating disorders.

## 2. Materials and Methods

The systematic review “The registration process is under processing” was conducted using different research databases, including PubMed, Cochrane, and Google Scholar. The search criteria were carefully selected to focus on particular aspects for the purpose of the study, and they included ‘Vitamin D’ [AND] ‘depression’ [OR] ‘epilepsy’ [OR] ‘bipolar’ [OR] ‘schizophrenia’ [OR] ‘neuroinflammation.’ By employing this targeted approach, the review was able to identify and synthesize evidence from multiple randomized controlled trials that examined the effects of vitamin D supplementation compared to placebos or no treatment. The studies covered a range of mental health and neurological conditions, providing a robust foundation to assess the potential therapeutic benefits of vitamin D.

### 2.1. Inclusion and Exclusion Criteria

The systematic review’s inclusion criteria for studies (summarized in Table 1) were primarily focused on two key aspects:

1. Vitamin D supplementation: The review specifically sought to examine research that investigated the effects of vitamin D supplementation on various health outcomes.
2. Specific mental health disorders: The included studies covered a range of mental health conditions, such as those outlined in the search terms—depression, epilepsy, bipolar disorder, schizophrenia, and neuroinflammation.

The systematic review specifically excluded certain types of studies from its analysis:

1. Studies that focused on mental or neurological disorders other than the key conditions of interest, namely depression, bipolar disorder, schizophrenia, and neuroinflammation.
2. Studies that examined the effects of vitamins other than vitamin D.
3. Studies that utilized multiple nutrients or supplements rather than focusing solely on vitamin D.
4. Studies that covered a range of mental health disorders rather than concentrating on the specific conditions outlined.
5. Comparative studies that did not include a vitamin D supplementation intervention.

All findings from the included studies were initially categorized according to the research question that guided the narrative analysis. However, due to the diverse range of research topics and analytical approaches employed across the studies, it was not feasible to provide a concise narrative summary that adequately captured the key findings from each individual study. As a result, a summary of all the studies is presented in the tables.

### 2.2. Search Strategy

**Figure 1:**
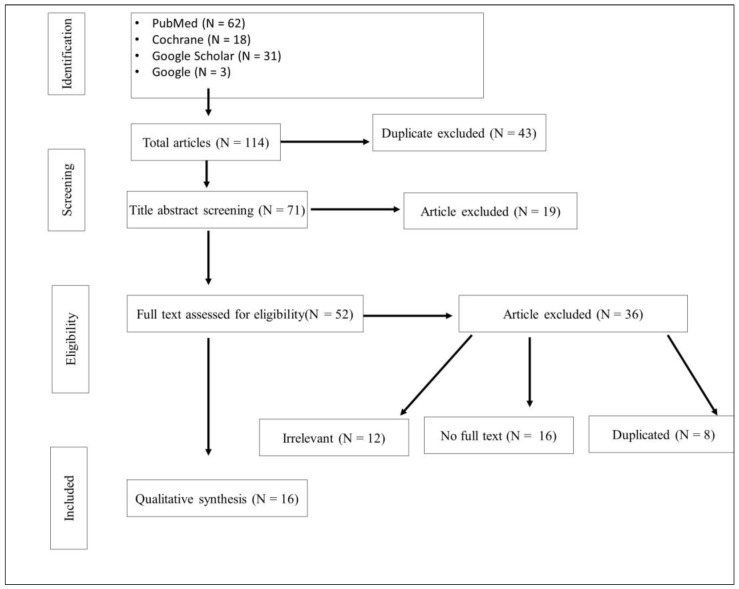
The inclusion procedure for the systematic review developed based on the PRISMA diagram [27].

The search strategy is summarized in Figure 1. It involved an electronic search through Google Scholar, the Cochrane database, and PubMed. The studies were analysed, and in case they did not meet the inclusion criteria or achieved specified exclusion criteria, they were disqualified from the systematic review.

### 2.3. Data Extraction

Each study was analysed, and specific information was extracted, including the authors, the study design, group characteristics such as age, type of disorder, vitamin D exposure, and the study findings (Table 2). To ensure the quality and reliability of the included studies and the overall conclusions drawn from the review, the Newcastle–Ottawa Scale (NOS) was incorporated to assess the quality and risk of bias.

## 3. Results

The key results are presented in Table 2 and Table 3 of the study. The review included a total of 16 studies examining the influence of vitamin D on the selected mental and neurological disorders: five studies focused on depression [28,29,30,31,32], three studies examined bipolar disorder [33,34,35], three studies looked at schizophrenia [36,37,38], four studies investigated epilepsy [39,40,41,42], and one study examined neuroinflammation [43].

Table 2 provides details on the study design, including the inclusion and exclusion criteria used to select the studies. Table 3 summarizes the key findings from each of these 16 studies. This allows for a high-level overview of the current research on the relationship between vitamin D status and these mental health disorders.

## 4. Discussion

Vitamin D is just as crucial for mental health as it is for physical health. While it is biologically plausible that vitamin D could play a role in depression, the data from human studies on this topic are limited. Some cross-sectional studies have found associations between lower levels of 25(OH)D (the main form of vitamin D in the body) and symptoms of depression [44], but other studies have not observed this relationship [8]. A systematic review and meta-analysis published in *The British Journal of Psychiatry* found that low levels of vitamin D were associated with a significantly higher risk of depression [45]. In contrast, the meta-analysis conducted by Gowda et al. did not find evidence supporting the effectiveness of vitamin D in improving depression symptoms among adults [32]. Similarly, the evidence from randomized controlled trials (RCTs) on the relationship between vitamin D and depression is inconclusive. The findings from different RCTs have revealed the beneficial effects of vitamin D supplementation on depressive symptoms [31], while other RCTs have not yielded similar results [28]. Another published study found that vitamin D supplementation resulted in significant improvements in depression scores in overweight and obese individuals [29]. Supporting these findings, our previous study published in 2020 examined the association between vitamin D and depressive symptoms in a sample of adults. We found that lower levels of vitamin D were associated with more severe depressive symptoms [14]. On the contrary, Alavi et al., in 2019, reported its lack of efficacy in treating depression in older adults [46]. The low levels of vitamin D in elderly individuals can be attributed to various factors, including reduced skin capacity to produce essential precursors for vitamin D synthesis, decreased exposure to sunlight, changes in adiposity, impaired renal function, and inadequate dietary intake [47]. Two of the studies in this review sufficiently supported that supplementing patients with vitamin D had an impact on depression. Patients considered in the studies proved this through a significant reduction in the Beck Depression Inventory (BDI) score [29,31]. Regardless, it is worth mentioning that while vitamin D supplements have shown benefits in alleviating depression and depressive symptoms, individuals with lower baseline levels of vitamin D may require higher serum levels to experience its antidepressant effects. This is particularly relevant for adults who have low vitamin D levels [30]. The effect of vitamin D on depression may be attributed to the fact that vitamin D is a unique neurosteroid hormone. As a neurosteroid, vitamin D has several important functions within the brain, including neuromodulation, supporting neurotrophic factors, providing neuroprotection, facilitating neuroplasticity, and contributing to brain development [48]. Vitamin D receptors are found on neurons and glial cells in various regions of the brain, such as the cingulate cortex and hippocampus [49]. Emerging research suggests that vitamin D deficiency may have been a significant contributing factor to stress-related depression during the COVID-19 pandemic [50]. Vitamin D is thought to influence the serotonergic system and help maintain healthy circadian rhythms, both of which are associated with depressive symptoms [50,51]. Overall, the literature suggests vitamin D may play a role in brain health and the development of depression, but the relationship is complex. More high-quality research is needed to clarify the effects of vitamin D on depressive symptoms and its potential therapeutic applications.

Bipolar disorder, another mental health condition, was also examined in relation to the effectiveness of vitamin D supplementation. The available data on the relationship between bipolar and vitamin D deficiency is inadequate. Marsh et al. conducted a small study, and despite the vitamin D supplementation group experiencing a greater increase in vitamin D levels, there was no significant difference in the reduction in depressive symptoms between the supplementation group and the placebo group. However, the vitamin D levels in both groups remained deficient overall. The vitamin D3 supplementation, when compared to placebo, did not improve the reduction in mood elevation or anxiety symptoms in bipolar patients [33]. Petrov et al. examined the relationship between vitamin D levels and inflammatory markers in a study involving 36 adolescents diagnosed with bipolar disorder. The study found that participants with bipolar disorder had significantly elevated levels of the vitamin D binding protein (DBP) compared to normal levels [35]. Previous studies have found that individuals with bipolar disorder exhibit elevated levels of circulating pro-inflammatory cytokines across different phases or stages of the disorder [52]. DBP has been suggested to act as a pro-inflammatory marker [53], so the higher concentration of DBP observed in bipolar patients might be attributed to the activation of the inflammatory process. Vitamin D has been found to have anti-inflammatory effects [54]. Therefore, it is possible that vitamin D might have a protective role in bipolar disorder by helping to reduce inflammation. Supporting the proposed protective role of vitamin D, a recent study on patients with bipolar disorder found that they exhibited significantly reduced levels of vitamin D [55]. Contrary to the previous findings, a recent study did not find significant differences between bipolar disorder patients and healthy controls in the serum concentrations of 25-hydroxyvitamin D [25(OH)D], 24,25-dihydroxyvitamin D [24,25(OH)2D], or the vitamin D metabolic ratio (VMR). Interestingly, the researchers did find an inverse correlation between the Young Mania Rating Scale (YMRS) scores and the levels of 24,25(OH)2D, as well as the VMR. Additionally, the prevalence of functional vitamin D deficiency and sufficiency was comparable between the bipolar disorder patients and the control group [56]. In conclusion, despite the consideration of vitamin D in the context of bipolar disorder, the available evidence suggests that conducting long-term assessments and evaluating patients across different phases of the disorder is still needed to provide more comprehensive insights into the relationship between vitamin D and the clinical course of bipolar disorder.

Regarding epilepsy, individuals with epilepsy frequently have inadequate levels of vitamin D, defined as a 25-hydroxyvitamin D3 concentration less than 20 ng/mL. Preclinical research and preliminary human data suggest that vitamin D3 may play a role in regulating or suppressing seizures through both membrane-based and genomic mechanisms [39,40,42]. Although the available evidence is inadequate, particularly in the context of adults, there have been studies examining the effects of vitamin D on epilepsy patients. A study by Hollo et al. found that in a group of 13 people with epilepsy (18–65 years old), there was an average 40% reduction in seizures after the subjects were given vitamin D supplementation. Notably, 38% of the participants experienced a 50% or greater reduction in their seizure frequency [40]. In addition, Alhaidari et al. demonstrated data that indicate low serum vitamin D levels were prevalent in 86.8% of the 542 people with epilepsy that were studied [39]. A study conducted in Egypt examined newly diagnosed paediatric patients with idiopathic generalized epilepsy. The results showed that 40% of these patients had vitamin D deficiency, and an additional 38% had vitamin D insufficiency [57]. Supporting these results, a recent study reported that 45.9% of the participants had vitamin D deficiency [58]. Mishra et al. have documented the importance of administering vitamin D supplements to children, highlighting the significant advantages such supplementation provides. These benefits include reducing seizures, regulating ionized calcium levels, and decreasing serum phosphate levels, thereby promoting the maintenance of healthy bones. These findings prove the necessity and effectiveness of vitamin D supplementation in these patients, ultimately mitigating the effects of the disease [41]. Since 1979, a substantial number of studies have highlighted the importance of vitamin D for people with epilepsy [59]. Many of these studies suggest that regular screening for vitamin D deficiency should be considered for individuals with epilepsy who are at risk [60,61]. Screening patients diagnosed with epilepsy for vitamin D deficiency regularly would allow for interventions that could help reduce the risk of future complications [62].

Vitamin D has long been linked to schizophrenia, but the majority of studies have faced challenges in reaching definitive conclusions regarding the efficacy of vitamin D and its impact on schizophrenia [36]. Vitamin D deficiency is very common in individuals with schizophrenia, particularly those experiencing acute episodes of the disorder [63]. Low levels of vitamin D in the blood may play a role in the development of schizophrenia, or schizophrenia and vitamin D deficiency may occur together due to shared genetic factors [64]. Valipour et al. have found a strong association between vitamin D deficiency and schizophrenia [37]. Contradictory, the results from two randomized controlled trials, one from Iran [38] and one from India [65], found that vitamin D supplementation did not lead to any changes in the symptom profile of patients with schizophrenia. Due to the inconclusive data, it remains uncertain whether supplementing vitamin D effectively reduces the symptoms associated with schizophrenia. While there is some evidence suggesting that vitamin D supplementation may improve cognition, there is no observed benefit or impact on psychosis, mood, or metabolic status [36]. The evidence from these studies suggests that vitamin D deficiency is not a causal factor in the pathogenesis of schizophrenia despite the high prevalence of vitamin D deficiency observed in schizophrenia patients. The relationship between the two appears to be more correlational than causational. Further research is still needed to determine if vitamin D supplementation is an effective treatment for patients with schizophrenia. This is especially important to investigate further, given the observed correlation between vitamin D deficiency and the occurrence of this mental health disorder, despite the lack of a proven causal relationship found in the studies described.

Lastly, the study also addressed the topic of neuroinflammation. The potential benefits of vitamin D have been extensively investigated in cell-based and animal studies of neurodegenerative diseases. Additionally, there are some clinical trial data and case reports exploring the effects of vitamin D supplementation in certain neurodegenerative conditions. According to the study conducted by Jiang et al., there is a strong association between vitamin D deficiency and a deficit in neuroinflammation in rats with traumatic brain injury [43]. The study’s conclusion stated that vitamin D therapy facilitated the shift of microglial cells towards the M2 phenotype, leading to reduced neuroinflammation. This effect was accomplished by inhibiting the TLR4/MyD88/NF-κB signalling pathway after traumatic brain injury. This confirms the substantial impact of vitamin D on neuroinflammation. Vitamin D deficiency may also be a risk factor for Alzheimer’s disease (AD). A meta-analysis conducted in 2019 found that individuals with severe vitamin D deficiency (<10 ng/mL) had a higher risk of developing AD and that every 10 ng/mL increase in vitamin D levels could reduce the risk of AD by 17%. However, these associations were not observed in those with vitamin D insufficiency (10–20 ng/mL). Animal research has further demonstrated that vitamin D deficiency in the early stages of AD can increase amyloid buildup in the hippocampus and cortex, as well as significantly inhibit cell proliferation, neurogenesis, and neuron differentiation in both normal and AD-like transgenic mice. Collectively, this evidence suggests that vitamin D deficiency may exacerbate the development and progression of Alzheimer’s disease [66,67,68]. Furthermore, another experimental study conducted on Male Wistar rats indicated that vitamin D has the potential to prevent or treat neurodegenerative diseases by suppressing the TLR4/MyD88/NF-κB pathway, which is known to play a crucial role in the development of myocardial inflammation [69,70]. Individuals with vascular dementia (VaD) have been found to have higher rates of vitamin D deficiency compared to the general population. This suggests that low vitamin D levels may be a risk factor for the development of vascular dementia. Conversely, recent research has indicated that higher vitamin D levels can help reduce the risk of developing vascular dementia [71]. While vitamin D has demonstrated efficacy in treating and preventing or ameliorating adverse effects associated with various mental and neurological illnesses, there are still certain conditions for which the benefits of vitamin D remain inconclusive. Examples of such conditions include schizophrenia and neuroinflammatory diseases.

## 5. Conclusions

There is a growing body of evidence suggesting a correlation between vitamin D status and various aspects of neurological and mental health, particularly in relation to conditions like depression and epilepsy. While the underlying mechanisms are still not fully understood, several mental and neurological disorders have demonstrated significant benefits associated with the use of vitamin D supplements. However, for some other health conditions, the impact of vitamin D has not been firmly established. Additional research is necessary to further investigate and clarify the potential benefits of vitamin D supplementation for those health disorders where the evidence remains inconclusive. In conclusion, the link between vitamin D and neurological/mental health is an area of increasing scientific interest and focus. More comprehensive studies are needed to fully elucidate the mechanisms by which vitamin D may influence brain function and the management of various neuropsychiatric conditions.

### Limitations

One key limitation of the existing systematic reviews on the relationship between vitamin D and neuropsychiatric conditions is the reliance on a relatively small pool of primary research studies. It is constrained by the currently limited number of high-quality, large-scale clinical trials investigating vitamin D’s effects on specific mental health or neurological outcomes. This can make it challenging to draw definitive conclusions as the evidence base may still be developing.

## Figures and Tables

**Table 1:** Patient, Intervention/Exposure, Comparator, Outcome, Study Design (PICOS) Criteria for the Inclusion/Exclusion Criteria.

Parameter	Inclusion Criteria	Exclusion Criteria
Population	Adults and children with specific mental health disorders (depression, bipolar and schizophrenia), and neurological disorders including (epilepsy and neuroinflammation)	Studies include other disorders, such as eating disorders and intellectual disabilities
Intervention	Vitamin D supplementation	Nutrients apart from vitamin D and other combined nutrients
Comparison	Influence of vitamin D on specified disorder assessed	No specificity in the comparison
Outcomes	Specific mental disorders including (depression, bipolar and schizophrenia), and neurological disorders including (epilepsy, and neuroinflammation)	Generalised mental or neurological health and comparison of different disorders in a single study.
Study design	Peer-reviewed studies published in English, including those that utilized; randomised controlled trials, retrogressive studies, randomised crossover trials, cohort studies, case-control studies, cross-sectional studies, systematic reviews, meta-analyses, and exception of experimental studies.	Articles not published in English, including websites, letters to the editor, expert opinions, case studies, case reports, and reports.

**Table 2:** Basic details and study design.

Author, Year	Study Design	Country (Location)	Study Group	Time
Depression				
Mousa et al., 2018 [28]	Cross-sectional study and randomised placebo-controlled trial.	Melbourne, Australia	Obese adults with BMI >25 kg/m^2^ from the age of 18 to 60 serum 25(OH)D concentrations <50 without clinical depression	Undefined
Jorde et al., 2008 [29]	Cross-sectional study and randomized double blind controlled trial	Norway	441 subjects (body mass index 28–47 kg m^−2^, 159 men and 282 women, aged 21–70 years)	Undefined
Sharifi et al., 2019 [30]	Double-Blind Randomised Placebo-Controlled Trial.	Shariati Hospital in Tehran	Patients above 35 years and with a BMI equal to and lower or higher than 25 Kg/m^2^	December 2014 and January 2015
de Koning et al., 2015 [31]	Randomised placebo-controlled clinical trial	Amsterdam, Netherlands	Older adults between 60 and 80 years.	June 2013 and April 2015
Gowda et al., 2015 [32]	Meta-analysis of Undefined. randomised controlled. trials	Undefined	Individuals aged ≥18 y who were diagnosed with depressive disorder	Up to 2014
Bipolar				
Marsh et al., 2017 [33]	Double-blind placebo-controlled trial.	Massachusetts, USA	18–70 years old with DSM IV bipolar Depression and Vitamin D deficiency (<30 ng/mL) Bipolar patients.	June 2013 and April 2015
Pirotta et al., 2015 [34]	Double-blinded, placebo-controlled randomized trial	Melbourne, Australia	Men and women (age > 60 with serum 25(OH) D concentrations	Undefined
Petrov et al., 2018 [35]	Longitudinal study	USA	A total of 36 participants (Youth aged 6–12) who were divided into three categories: non-mood controls (n = 13), bipolar disorder (BD) (n = 12) and major Depressive Disorder (MDD) (n = 11)	Undefined
Schizophrenia				
Krivoy et al., 2017 [36]	Randomized, Double-Blind, Placebo-controlled Clinical trial	Geha Mental Health Center, Israel	Schizophrenia patients who had been maintained on clozapine treatment for at least 18 weeks and had low levels of vitamin D (<75 nmol/L)	1 May 2014 to 30 September 2016.
Valipour et al., 2014 [37]	Systematic Review and Meta-Analysis of Observational Studies	Undefined	Patients with schizophrenia	Up to October 2013
Sheikhmoonesi et al., 2016 [38]	Randomized controlled trial	Iran	Patients aged 18 to 65 years who met the Diagnostic and Statistical Manual of Mental Disorders, Fourth Edition, Text Revision (DSM-IV-TR) criteria for a diagnosis of schizophrenia. All participants had baseline serum vitamin D concentrations less than 30 ng/mL, indicating either vitamin D deficiency (less than 20 ng/mL) or vitamin D insufficiency (21 to 29 ng/mL).	March 2013–September 2013
Epilepsy				
Alhaidari et al., 2022 [39]	Retrospective analytical medical record review	Outpatient epilepsy research clinic in Saudi Arabia	Epileptic patients older than 14 years.	November 2016 and April 2020
Hollo et al., 2012 [40]	Pilot study	Budapest, Hungary	Patients between 19 years and 60 years.	Undefined
Mishra et al., 2023 [41]	Randomised Controlled Trial	Undefined	Children aged 2 to 12 years old with epilepsy.	Undefined
Al Khalifah et al., 2018 [42]	Randomised pragmatic trial protocol	Paediatric neurology clinic at King Saud University Medical City, Saudi Arabia.	Children aged 2 to 16 years with epilepsy.	December 2017 and December 2019
	Neuroinflammation			
Jiang et al., 2022 [43]	Experimental study (behavioural tests in traumatic brain injury rats)	Beijing, China	Sprague-Dawley (SD) rats (age, 12–16 weeks, weighing 280–320 g).	Undefined

**Table 3:** Results and findings summarised from the studies included accompanied by Newcastle–Ottawa Scale (NOS).

Author, Year	Outcome	Conclusion from Studies	Quality Based on NOS
Depression			
Mousa et al., 2018 [28]	No significant reduction in Beck Depression inventory score in patients included in the study.	Non-supporting	6
Jorde et al., 2008 [29]	Overweight and obese subjects with serum 25(OH)D levels <40 nmol L^−1^ have higher (more depressive) scores on the BDI. A significant improvement BDI scores.	Supporting	6
Sharifi et al., 2019 [30]	Patients with sufficient baseline vitamin D may benefit from supplementation more than vitamin D-deficient patients, which indicates that higher serum vitamin D levels may be needed for its antidepressant effect.	Supporting	8
de Koning et al., 2015 [31]	Supplementation with 1200 IU/d vitamin D for 12 months did not affect depressive symptoms and physical functioning in older persons with relatively low vitamin D status.	Supporting	6
Gowda et al., 2015 [32]	The study did not support the evidence for the efficacy of vitamin D in improving depression among adults.	Inconclusive	6
Bipolar			
Marsh et al., 2017 [33]	Despite a more significant rise in Vitamin D levels in the Vitamin D supplementation group, there was no significant difference in reducing depressive symptoms. Additionally, Vitamin D3 supplementation vs. placebo did not improve reduction in mood elevation or anxiety symptoms.	Non-supporting	7
Pirotta et al., 2015 [34]	Daily supplementation with 2000 IU of vitamin D3 for ten weeks had no significant effect on neuroplasticity compared to placebo, but the finding that vitamin D treatment alone was associated with a decrease in corticospinal excitability and intracortical inhibition.	Inconclusive	6
Petrov et al., 2018 [35]	Increased levels of Vitamin D Binding Protein (DBP) in participants with bipolar disorder (BD) compared to the non-mood control group.	Supportive	6
Schizophrenia			
Krivoy et al., 2017 [36]	Providing vitamin D supplements did not demonstrate a clear superior benefit compared to the placebo treatment when evaluating the primary outcomes related to psychotic, depressive, and metabolic outcomes. A mild positive effect of vitamin D supplementation seen on cognitive performance in the participants.	Inconclusive	7
Valipour et al., 2014 [37]	A strong association between vitamin D deficiency and schizophrenia.	Supportive	6
Sheikhmoonesi et al., 2016 [38]	No relationship between serum vitamin D level changes and the improvement of negative and positive symptoms in schizophrenic patients	Not supporting	6
Epilepsy			
Alhaidari et al., 2022 [39]	Patients with high vitamin D levels had reasonable seizure control compared with those with low levels.	Inconclusive (Recommended RCT)	6
Hollo et al., 2012 [40]	Supplementation of vitamin D had a significant impact on decreasing seizures.	Supporting.	7
Mishra et al., 2023 [41]	Supplementation of vitamin D had a significant impact on decreasing seizures.	Supporting.	7
Al Khalifah et al., 2018 [42]	The results proved the efficacy of maintaining higher vitamin D levels and its impacts on seizure control and bone health for children with epilepsy.	Supporting.	6
Neuroinflammation			
Jiang et al., 2022 [43]	Vitamin D effectively alleviated neurocognitive deficits, brain oedema, and blood brain barrier disruption, and promoted hippocampal neuronal survival in vivo and in vitro.	Supporting	-
